# Living better or living longer? Perceptions of patients and health care professionals in oncology

**DOI:** 10.3332/ecancer.2015.574

**Published:** 2015-09-22

**Authors:** Diego de Araujo Toloi, Gabriela Critchi, Andrea Mangabeira, Felipe Matsushita, Rachel P Riechelmann, Paulo M Hoff, Everardo D Saad

**Affiliations:** 1 Instituto do Câncer do Estado de São Paulo, Avenida Doutor Arnaldo, 251, São Paulo, Brazil; 2 Faculdade de Medicina da Universidade de São Paulo, Avenida Doutor Arnaldo, 455, São Paulo, Brazil; 3 Dendrix Research Ltd, Rua Joaquim Floriano, 72/24, São Paulo, Brazil

**Keywords:** cancer, overall survival, quality of life, treatment

## Abstract

**Background:**

Cancer can influence the views of patients on treatment goals and make them different from those of health care professionals (HCPs). It is crucial to understand patient expectations regarding cancer treatment.

**Methods:**

We performed a cross-sectional survey of patients with cancer and HCPs to evaluate their perceptions about treatment priorities and to analyse variables that might influence their opinions. To identify treatment choices, we interviewed all participants using a structured questionnaire with fictitious case vignettes.

**Results:**

We enrolled a total of 230 participants, including 144 patients and 86 HCPs (35 nurses, 21 physicians, 30 others). Treatment priority between survival time (28.5% for patients vs. 8.1% for HCP) and quality of life (45.8% vs. 87.2%) differed significantly, with the remaining participants stating they were uncertain or unwilling to respond, or providing invalid responses (*P* < 0.01). In logistic regression, prioritising survival time was more frequent in patients, adjusting for age and gender (odds ratio (OR) = 3.95; *P* < 0.01). The view that the physician alone should be responsible for treatment choices was more frequent among patients than HCPs (18.8 vs. 5.8%; *P* = 0.01).

**Conclusions:**

In Brazil, our results suggest that survival time is more important for patients with cancer than for HCPs, whereas quality of life is more important for HCPs than for patients with cancer, who place great emphasis on physicians as decision-makers. Given that Brazilian patients often rely on physicians for decisions, the potential impact of different priorities between survival time and quality of life when patients and HCPs are compared is unknown.

## Introduction

The increasing spectrum of available therapies, with potentially different benefits and risks of adverse events, as well as the heterogeneous profile of patients with cancer, make therapeutic decision-making a challenge for both patients and medical oncologists. The so-called ‘clinical benefit’ can have different meanings in clinical practice when discussing treatment planning [1]. Sharing decisions, taking into account one’s personal and family values and expectations, may be complex. In this regard, there has been increasing interest in understanding the perceptions of cancer patients about treatment goals, such as extending the survival time of and/or prioritising quality of life.

Several studies have looked at patients’ expectations from treatment [2, 3, 4]. A US study with 181 women with metastatic breast cancer showed that patients highly valued overall survival even if the potential gains came at the cost of more side effects [2]. Patient-related characteristics, which may influence treatment decisions, are also important to be evaluated. A study conducted in the Netherlands showed that anxious/depressed patients had a higher probability to opt for radical treatment for early prostate cancer [3]. Expectations from treatment also relates to patient understanding of the disease. A prospective US study with 917 patients with Stage III or IV non-small-cell lung cancer or colon cancer reported that patients who considered themselves to have a better prognosis favored life-extending therapies [4]. It is conceivable that patients’ opinions vary according to other features, such as country of origin, religion, or social status. Moreover, patients with different characteristics may rely on their physicians for treatment decisions to differing extents. For example, a multicenter study from Iran showed that while 85.2% of 980 patients with cancer were willing to receive detailed information about their disease, 56.9% preferred to leave decisions up to their physician [5]. There is limited information about the treatment expectations of patients with cancer in Brazil, and little is known about the influence of patient characteristics.

We aimed to evaluate the treatment priorities of both patients with cancer and health care professionals by comparing their preference regarding extension of survival time or maintenance of quality of life. Additionally, we wanted to identify possible factors that might influence these choices and to assess patients’ perceptions about who should be the main players in their treatment decision-making.

## Methods

### Study design

We performed a cross-sectional study of patients with cancer and health care professionals at one of the largest publicly funded cancer hospitals in the country. Eligible participants were 18 years of age or older, were either patients who had been diagnosed with cancer for at least 6 months and who were under active anticancer therapy (patient group), or were HCPs working at the same hospital (HCP group). Participants were excluded if they had any known major psychiatric illness or cognitive dysfunction. The study was approved by our local institutional review board, and written informed consent was obtained from all participants.

### Data collection

Demographic and social data (age, gender, level of education, marital status, family cancer history, and religion) were collected from all participants as part of the questionnaire. For patients, information on tumor type and treatment characteristics was retrieved from medical charts. All information was placed in a database and was treated confidentially.

We used a slightly modified version of a structured questionnaire with fictitious case vignettes developed for a previous study conducted by our group [6]. Participants were approached by one of the authors (DAT or GC) during treatment days and were given enough time to read the informed consent document before completing the questionnaire (in Portuguese). No demographic or clinical data were collected for the few potential participants who refused to be enrolled, nor was the reason for such refusal inquired. Based on the vignettes, participants were asked to give their opinion about the treatment priorities for each fictitious case. Additionally, they were asked to indicate who they thought should have more weight in the treatment–decision process. The treatment priority regarding survival time versus quality of life was assessed in a closed question: “a patient has just discovered that he/she has cancer. The doctor discusses about the types of treatment and possible side effects. In your opinion, what treatment priority should be chosen?” That question allowed only one answer, with the three options being: “the kind that provides a longer survival time, regardless of quality of life”, “the kind that provides the maximum quality of life, although the survival time is not prolonged by treatment”, and “I do not know or prefer not answer”. The answers were considered invalid when patients indicated more than one alternative. Treatment priority was also assessed using four-case vignettes for fictitious patients at four different ages: 5 years, 16 years, 50 years, and 70 years, and an explanation was requested for each case with an open question.

The decision-making process was assessed with the closed question: “a patient has just discovered that he/she has cancer. Who, in your opinion, should be involved in treatment choices?” Participants could mark one or more of the following options: the physician, the patient, the family, and other member of the health care team.

Investigators were responsible for implementing the questionnaire, for checking its completion and ensuring data quality.

### Statistical analysis

The total sample size was pre-defined as 200 participants (100 in each group). The sample size was determined based on feasibility rather than on statistical assumptions. This number was also based on our previous experience in a similar survey conducted in another setting [6].

Summary statistics were used to describe participants’ characteristics and responses. Comparisons of characteristics between and within patients and HCPs were made according to the nature and distribution of statistical variables of interest. Numerical variables were compared by Student’s *t*–test or Mann–Whitney U-test, while categorical variables were compared by the Chi-square or Fisher’s exact tests. Multivariate analysis was performed using logistic regression models, in order to assess the association between independent variables and categorical outcomes of interest. The analyses were conducted using MedCalc software (Mariakerke, Belgium, version 11.0.0), with two-tailed significance level of 5%.

The answers to open question to the four case vignettes were submitted to categorisation for descriptive analysis.

## Results

From January 2013 to March 2014, 230 participants were enrolled (144 patients and 86 HCPs). The main characteristics of the participants are detailed in Table 1. The group of patients had a higher median age (53 vs. 29 years; *P* < 0.01) and a lower proportion of females (59.7% vs. 74.4%; *P* = 0.03) than the group of HCPs. Patients more often had gastrointestinal (40.3%), breast (20.8%), or lung (6.9%) tumors, and tumor-node metastasis (TNM) stages were I to III (40.3%), IV (58.3%), or unknown (1.4%).

### Treatment priorities

**Figure 1.**
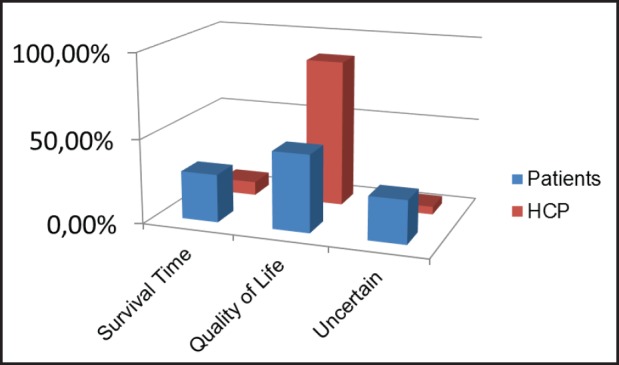
Priority treatment question. HCP = health care professionals.

Figure 1 shows the distribution of responses to this item of the questionnaire. Treatment priorities for the 144 patients were survival time in 28.5% (*N* = 41), and quality of life in 45.8% of cases (*N* = 66); 20.1% of patients were uncertain or unwilling to respond (*N* = 29); whereas an invalid response was given in 5.6% cases (*N* = 8). Treatment priorities for the 86 HCPs were survival time in 8.1% (*N* = 7), and quality of life in 87.2% of cases (*N* = 75); only 3.5% if HCPs were uncertain or unwilling to respond (*N* = 3), and an invalid response was given by only one individual (1.2%). There was a statistically significant difference between the distribution of treatment priorities between the two groups (*P* < 0.01). In univariate analyses, age was significantly (and positively) associated with prioritising survival time (*P* < 0.01), whereas no similar association was found for gender. In multivariate logistic regression, prioritising survival time was more frequent for patients than for the HCPs after adjustments were made for age and gender (OR, 3.95; *P* < 0.01) (Table 2). Age was not predictive of prioritising survival time in multivariate analysis. Nearly, identical results were obtained in a model with only age and participant group as independent variables (data not shown).

Among patients, prioritising survival time did not differ significantly between Stages I to III versus IV. Among HCPs, prioritising survival time did not differ significantly when nurses, physicians and others were compared.

While patients prioritised the choice of treatments with the greatest chance of cure, HCPs tended to choose less aggressive treatments that could offer better quality of life for 50 years and 70 years cases (Table 3). The main reasons given by participants, in a total of 920 responses (four questions justified by a total of 230 participants, with 576 patient responses, and 344 HCP responses) regarding their choice of treatment were factors associated with treatment itself (effectiveness, toxicity, and duration) in 45.31% of patient responses and 82.85% of HCP responses, and factors related to the patient’s clinical condition (life expectancy, performance status) in 36.46% of patient responses and 57.27% of HCP responses.

### Role in decision-making

**Figure 2.**
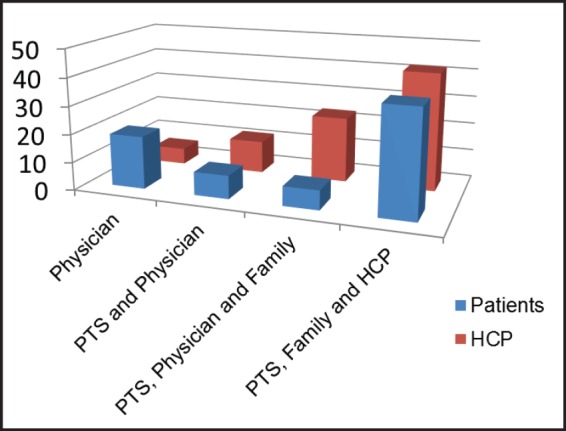
Decision-making question. PTS = patients; HCP = health care professionals.

The main responses indicated in the question about the process of treatment decision-making are summarised in Figure 2. The statement that ‘the physician alone should be responsible for treatment choices’ was more frequent among patients than among HCPs (18.8% vs. 5.8%, *P* = 0.01). Only 33.3% of patients said they would be willing to be involved in the decision about their own treatment.

## Discussion

In this cross-sectional survey using fictitious case vignettes, we observed that the overwhelming majority of oncology HCPs and nearly half of patients prioritised treatments that offered better quality of life. However, patients with cancer were more likely to opt for treatments that prolong their survival than HCPs. Among patients, opinions were not significantly influenced by gender or tumor stage. The proportion of patients who considered that the physician alone should be responsible for treatment decision–making was higher when compared with that of HCPs.

A British study published in 1990 with 1,441 participants found that patients with cancer were more likely to accept more treatments with minor chance of cure or prolonging life or relief of symptoms than the group of cancer specialists and general practitioners [7]. Another British study, published in 2001, with 545 participants, showed that patients accepted a lower chance of benefit than healthy controls and the health care team (medical and clinical oncologist, palliative care and nursing) in a second-line chemotherapy scenario [8]. A Japanese study with 73 patients with lung cancer showed when the chance of symptom relief was 70%, 73% of patients were willing to choose intensive chemotherapy [9]. A recent review of literature [10] reinforces these results [7, 8, 9]: patients tend to prioritise survival to a greater extent than HCPs or well people. On the other hand, a US survey of 1,000 patients with prostate cancer showed that patients were more willing to preserve quality of life (45%), whereas more than 90% of 200 urologists preferred extending patient survival [11]. Of note, patients that participated in this survey belonged to a prostate cancer support group, and the extent to which this feature may influence the study results is unclear.

A similar study was conducted by the same team in a Brazilian private hospital with patients, HCP and laypersons, investigating treatment priorities for cancer in the private-care setting [6]. The results suggested that survival time was also a higher priority to people who were diagnosed with cancer, whereas quality of life appeared to be of higher priority to HCPs. Brazil has a dual health care system, with patients treated at private institutions having access to a broader range or reimbursed medications. Indeed, one could think that patients treated in the private setting could be more willing to receive therapies that extended their survival because of the availability of such treatments. On the other hand, patients treated in the public system, where resources are limited and their social economic status is lower, could be more likely to receive less aggressive interventions. Of note, the profile of HCPs of private and public institutions appears to differ less than that of patients, and indeed there is overlap in medical staff between the institutions assessed in the previous and in the current survey (although none of the current participants took part in the previous survey). Interestingly, the present study demonstrated that patients with cancer from both settings prioritised life extension more often than HCPs, whereas the latter prioritised quality of life more often than patients.

The evidence that patients prioritise survival time to a greater extent than physicians bears potential implications in clinical trials. Probably, the primary endpoint of overall survival or a surrogate efficacy endpoint may be more attractive than studies that aim at solely improving quality of life or other endpoints. However, this might change according to the presence of symptoms. For example, in tumor types, where there is a great symptom burden, the use of patient-reported outcomes, such as quality of life, may still be attractive to patients. Indeed, the Prostate Cancer Working Group 2 consensus 2008 recommended that clinical trials used outcomes to control, relieve or eliminate sign and symptoms, and to prevent or delay future events, such as skeletal related [12].

Another important point that may influence patients’ perspective on treatment goals is longevity. Longer life expectancy has led to increased incidence of cancer in the older population. While we speculated that older patients could have different views on treatment goals, age was not associated with survival priority in our study.

An important issue to take into account in assessing the decision-making process is the amount and the quality of information given to patients and families about diagnosis, prognosis, and treatment. A systematic review of communication in cancer care has shown that most patients want prognostic information, as well as they value help and support from their physicians concerning information on prognosis and maintain a therapeutic alliance [13]. The majority of the studies were conducted in early-stage cancer settings, what highlights the lack of evidence about prognosis communication on different scenarios throughout the course of a patient with cancer [13]. In our survey, we observed that patients with cancer often rely on physicians for treatment decisions, in a somewhat paternalistic fashion [14]. Such attitudes are influenced by social and ethnic characteristics, as demonstrated in the case of the ‘latino’ culture [15], which is prevalent in Brazil. Potential factors further influencing the decision-making process include lack of proper understanding of the disease and available treatments, emotional weakness of the patients, and denial of diagnosis or prognosis.

Our study has some limitations. First, this was a cross-sectional view of the dynamic process of cancer therapy. It is expected that patients’ perceptions may change throughout the course of the disease, notwithstanding the indirect evidence of no significant difference in treatment priorities according to TNM stage. A second limitation is the study size, which is relatively small in comparison with some of the previous surveys on this subject. On the other hand, our study was larger than some of the published surveys of this type, and the inclusion of a sizable group of HCPs gave the study enough power for some of the major comparisons of interest. There was also a possible selection bias because the median age of HCPs was only 29 years. Although logistic regression was used to adjust for such imbalance, it is possible that residual confounding still remained after this adjustment, as young, less experienced professionals put less emphasis on survival because they are, theoretically, further away from death. It should be noted that our results may not be applicable to patients with haematological malignancies, many of whom are young adults that undergo treatments that are very aggressive but often have a curative intent.

## Conclusion

In conclusion, we found that treatment priorities differ significantly between patients with cancer and HCPs in Brazil. Like patients with cancer from other countries, we found that Brazilian patients more often prefer treatments that extend their survival than HCPs, the latter focusing on quality of life to a greater extent than patients. Moreover, Brazilian patients appear to rely on physicians for the decision-making process during treatment. The extent to which different priorities within these two groups may lead to treatment decisions that are not in accordance with patient priorities remains unknown and is an important topic for future studies. Prospective studies would be particularly useful, since decision-making is a dynamic process that may change throughout the course of cancer therapy. Finally, the information of this type is quite relevant for drug development in oncology, as investigators aim at designing clinical trials and using endpoints that are in line with patient and public expectations.

## Compliance with ethical standards

The authors declare that they have no conflict of interests regarding the publication of this article. The study was approved by our local institutional review board, and written informed consent was obtained from all individual participants included in this study. All procedures performed were in accordance with the 1964 Helsinki Declaration and current ethical and regulatory standards in Brazil.

## Figures and Tables

**Table 1.** Characteristics of the participants.

	Patients (*N* = 144)	Health care professionals (*N* = 86)
Age, years – Median (Range)	53 (17–82)	29 (22–57)
**Sex (%)**		
Female	59.7%	74.4%
Male	40.3%	25.6%
Married – no. (%)	77 (53.47%)	33 (38.37%)
With children – no. (%)	107 (74.3%)	22 (25.58%)
Family history of cancer – no. (%)	101 (70.13%)	67 (77.9%)
**Cancer type**		
Gastrointestinal	58 (40.3%)	
Breast	30 (20.8%)	
Lung	10 (6.95%)	
Other	46 (31.95%)	
**TNM Stage**		
I	2 (1.4%)	
II	12 (8.3%)	
III	44 (30.6%)	
IV	84 (58.3%)	
Unknown	2 (1.4%)	
**Current treatment**		
Neoadjuvant	12 (8.3%)	
Adjuvant	34 (23.6%)	
Palliative	86 (59.8%)	
In follow-up	11 (7.6%)	
Unknown	1 (0.7%)	
**Health care professionals**		
Physician		21 (24.4%)
Nurse		35 (40.7%)
Psychologist		14 (16.3%)
Pharmacist and nutritionist		16 (18.6%)

**Table 2.** Results of logistic regression for prioritising survival time.

Variables	Odds ratio	95% CI^a^	*P* value
Age	0.99	0.97–.02	0.63
Gender	1.04	0.53–2.07	0.91
Group (HCP^b^ as reference)	3.95	1.43–10.89	<0.01

a CI = confidence interval

b HCP = health care professionals

**Table 3.** Priority treatment question by age of fictitious cases.

3A. Patients				
	Treatment X	Treatment Y	Treatment Z	Not answered or marked more than one
5 years	73.62%	14.58%	6.25%	5.55%
16 years	72.23%	17.36%	4.16%	6.25%
50 years	65.28%	17.36%	9.03%	8.33%
70 years	38.19%	29.17%	20.84%	11.80%

3B. Health care professionals				
	Treatment X	Treatment Y	Treatment Z	Not answered or marked more than one
5 years	86.05%	4.65%	9.30%	0%
16 years	89.53%	5.82%	4.65%	0%
50 years	53.49%	32.56%	13.95%	0%
70 years	19.76%	15.12%	65.12%	0%

**Treatment X:** The treatment is quite toxic and the person needs to be hospitalised for approximately 1 month to recover. The visits will be restricted due to low resistance of the patient. The chance of cure is high.

**Treatment Y:** The treatment can cause nausea, vomiting, fever, and tremors, but is less toxic than treatment X. This treatment should be administered in the hospital once a week for 1 year without need for hospitalisation. This treatment will not cure the patients, but can prolong life by some months.

**Treatment Z:** The treatment should be administered once a month. For each administration, the patient has to stay for 30 minutes in the hospital. The most common side effects are fairly mild, but this treatment is probably less effective than treatment Y.
